# Matrix Metalloproteinases in Inflammatory Dermatoses

**DOI:** 10.3390/ijms262110319

**Published:** 2025-10-23

**Authors:** Joanna Czerwińska, Agnieszka Owczarczyk-Saczonek

**Affiliations:** Department of Dermatology, Sexually Transmitted Diseases and Clinical Immunology, School of Medicine, Collegium Medicum, University of Warmia and Mazury in Olsztyn, 10-719 Olsztyn, Poland

**Keywords:** metalloproteinases, inflammatory skin disorders, atopic dermatitis, psoriasis, scleroderma, blistering diseases, alopecia areata, prurigo nodularis

## Abstract

Matrix metalloproteinases (MMPs) are endopeptidases that help maintain tissue homeostasis. Dysregulation of MMP secretion or activity, along with issues in their natural regulators, contributes to the development of many disorders, including autoimmune skin diseases. This article provides a comprehensive review of current research on MMP biology, their physiological functions, and disease-specific evidence in dermatology. Based on available English-language studies, we discuss key papers with important findings and the latest systematic reviews from PubMed. Additionally, a comparison, synthesis, and summary of reported results are included to highlight the specific role of MMPs in dermatology and to identify research gaps that need to be addressed for developing and using MMPs as potential biomarkers in skin disease pathophysiology.

## 1. Introduction

### Physiological Role of Matrix Metalloproteinases in the Skin

The family of matrix metalloproteinases (MMPs), which depends on biological structures, includes six subtypes of endopeptidases: astacins, adamalysins (ADAMs—containing a disintegrin domain, metalloproteinase domain, often an EGF-like domain, and a short cytoplasmic tail, mainly associated with the cell surface or membrane) [1], ADAMTS with thrombospondin motifs, pappalysins, serralysins, and classical MMPs [2,3,4,5,6]. These enzymes are produced inside cells and then released in an inactive form (proMMP), regulated by zinc ions [7], into the extracellular space during the formation of extracellular networks (ET) [8].

The primary function of MMPs is to maintain tissue homeostasis by preserving structural integrity and enabling responses to changing conditions, making them especially important in dermatology [9]. They control cellular behavior and connective tissue composition through both intracellular signaling and direct remodeling of extracellular matrix (ECM) components. The key parts of the ECM include collagens, proteoglycans, and glycoproteins, which help maintain proper tissue structure. Associated proteins like growth factors and cytokines mainly regulate these processes [2]. The ECM is produced and adjusted by fibroblasts, giving the skin stability, mechanical strength, and elasticity. Due to MMP activity, the makeup and features of skin ECM are constantly changing—through synthesis or breakdown—during embryonic development, growth, maturation, and in disease states. Also, ECM proteolysis can release bound factors or form bioactive fragments that influence processes such as cell migration, chemotaxis, and proliferation. Proteolytic processing can happen at the cell surface (or membrane receptors) or in the extracellular space [3,8,9].

Under normal conditions, MMPs—particularly certain key types—are involved in many biological processes, including embryogenesis, morphogenesis, reproduction, wound healing, and ion metabolism regulation [10]. Their activity is tightly regulated both at the gene level (since different cell types can produce varying amounts of MMPs depending on stimuli) [11,12] and at the protein level, through translation and natural inhibitors like α2-macroglobulin and tissue inhibitors of metalloproteinases (TIMPs) [13,14]. It is important to note that most MMPs are either produced in small amounts or remain inactive under normal conditions. In some cases, slightly higher MMP levels can actually protect against disease. MMP production is controlled by chemokines, cytokines, and growth factors, and is also affected by cellular stress [15].

## 2. Disorders in the Activity of Metalloproteinases in the Skin

**Figure 1:**
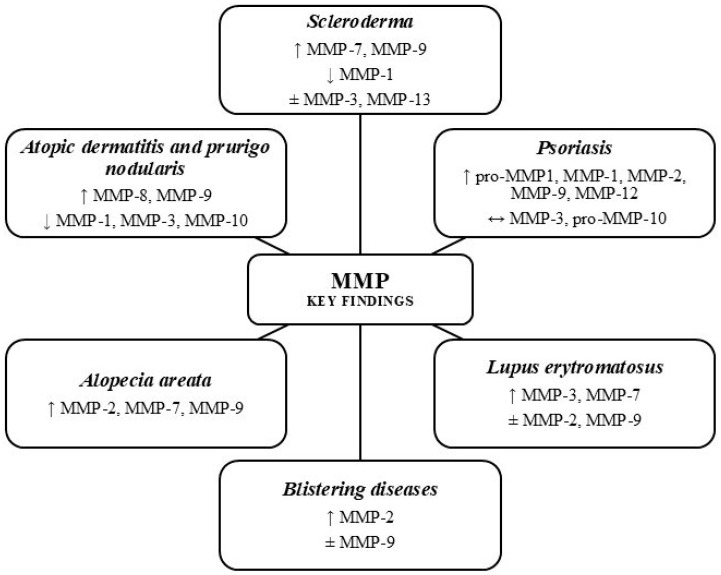
Key findings from studies on MMP expression in selected inflammatory skin diseases. ↑ Increased, ↓ Decreased, ↔ No Change, or ± Conflicting Data. Diagram prepared based on sources [20,21,22,23,24,25,26,27,28,29,30,31,32,33,34,35,36,37,38,39,40,41,42,43,44,45,46,47,48,49,50,51,52,53,54,55,56,57,58,59,60,61,62,63,64,65,66,67,68,69,70,71,72,73,74,75,76,77,78,79,80,81,82,83,84,85,86,87,88,89,90,91,92].

Disruptions in the secretion and activity of various types of MMPs, which are linked to changes in ECM deposition and breakdown [2], may play a key role in the development of many diseases. In the field of MMPs, most focus has been on their role in cancer progression, including melanoma [16]. The secretion and activity of MMPs are increased in nearly all types of human cancers. Additionally, a link has been shown between MMP levels and tumor stage, invasiveness, metastatic ability, and shorter survival times [17,18]. The most significant roles are played by MMP-2 and MMP-9, which break down type IV collagen, the primary component of the basement membrane [6]. Disruptions in tissue balance caused by altered MMP activity or their natural inhibitors directly or indirectly help cause other health issues, such as inflammatory responses, degenerative conditions, rheumatic disorders, cardiovascular problems, lung diseases, and autoimmune disorders [19]. Key points regarding MMPs in the presented inflammatory skin disorders are summarized in Figure 1.

- Scleroderma

Systemic sclerosis (SSc), caused by its fibrotic pathogenesis driven by excessive collagen synthesis, was one of the first diseases in which MMPs were widely studied. Both gene and protein analyses consistently show reduced MMP-1 activity [20,21,22,23]. This downregulation seems to be affected by hepatocyte growth factor (HGF) [20] and *miR-202-3p* expression [21]. Experimental studies also suggest that rapamycin has antifibrotic effects by increasing *MMP-1* transcription [22], whereas phototherapy does not significantly change MMP-1 levels despite clinical improvements [23].

Other MMPs show disease-specific patterns. MMP-13 expression is decreased in generalized morphea but unchanged in other subtypes [24]. MMP-3 may counter fibrosis by degrading α2-antiplasmin (α2AP) [25], whereas MMP-9 and MMP-14 levels are elevated in diffuse cutaneous SSc [26,27]. Autoantibodies against MMP-1 were detected in both localized scleroderma and SSc, especially in morphea, and were associated with higher anti-nucleosome antibody levels and shorter disease duration [28].

Clinical correlations suggest that MMP/TIMP ratios may reflect specific manifestations of scleroderma. Although serum MMP-3 often remains unchanged [25,29], it has been proposed as a potential predictor of rheumatoid arthritis overlap in SSc patients [30]. In localized scleroderma, reduced MMP-13 correlates with muscle involvement but fewer skin lesions [31].

Genetic studies show that polymorphisms in *MMP-1* and *MMP-3* are linked to distinct clinical phenotypes: for example, the *MMP-1 1G/1G* genotype correlates with interstitial lung disease, while the *MMP-3 5A/5A* variant is associated with anti-topoisomerase antibodies [32]. *MMP-9 rs3918242* may protect against digital ulcers, particularly in males [30]. Elevated MMP-7 levels correlate with worse pulmonary function in SSc-ILD [33,34], and combined serum MMP profiles may aid in early, noninvasive diagnosis of ILD in SSc and RA [32]. In addition, increased MMP-9 and inflammatory chemokines such as IL-6 and CXCL4 have been observed in SSc patients with periodontal disease [35].

Overall, MMP dysregulation in systemic sclerosis reflects a complex imbalance between decreased MMP-1 and MMP-13 (profibrotic suppression) and increased MMP-9, MMP-14, and MMP-7 (proinflammatory activation). This indicates that different MMP patterns could act as both mechanistic markers of tissue remodeling and potential biomarkers for organ-specific complications.

b. Psoriasis

Studies on MMPs in psoriasis have been conducted at both the gene expression [36] and protein [37] levels across various subtypes, including plaque and pustular psoriasis. Genetic analyses demonstrated increased expression of *MMP-1* and *MMP-12* [36], as well as *MMP-2* and *MMP-9* [37,38], in lesional skin. Moreover, *MMP* expression was found to increase following IL-17 stimulation—both in cultured keratinocytes and in psoriatic lesions [39]. Notably, MMP-9 mRNA expression in non-lesional skin of patients with pustular psoriasis was higher than in those with plaque psoriasis [38].

Serological studies by Flisiak et al. revealed elevated serum levels of MMP-1 and TIMP-1, while MMP-1 content in psoriatic scales negatively correlated with disease severity, as assessed by PASI [40]. Treatment-response data further support the involvement of MMPs in psoriasis pathogenesis: in patients receiving anti-TNF-α therapy, reductions in MMP-9 levels were observed in PBMCs, serum, and skin, paralleling clinical improvement [41]. Similarly, Michalak-Stoma et al. (2021) reported increased serum levels of pro–MMP-1 and MMP-9, while MMP-3 and pro–MMP-10 showed no significant differences compared to healthy controls [37].

Animal model studies demonstrated that neutrophils and neutrophil extracellular traps (NETs) are key sources of MMPs in psoriasis, contributing to vascular endothelial cell (VEC) activation, increased vascular permeability, and angiogenesis. Pharmacological inhibition of MMP-9 reduced inflammatory symptoms [42]. Interestingly, comparisons between psoriatic plaques and atherosclerotic lesions revealed dysregulated expression of MMP-2 and MMP-9 in both conditions. MMP-9 was elevated in both psoriatic and atherosclerotic tissues, whereas MMP-2 showed the opposite trend—reduced in psoriatic skin but elevated in atherosclerotic plaques [43,44].

In summary, patients with psoriasis exhibit increased expression of MMP-1, MMP-2, and MMP-9 at both the gene and protein levels, partly correlating with inflammation intensity. MMP-9 was elevated in both psoriatic and atherosclerotic tissues, whereas MMP-2 showed the opposite trend—reduced in psoriatic skin but elevated in atherosclerotic plaques.

c. Lupus erythematosus (LE)

Unlike the well-established role of MMPs in psoriasis, their involvement in lupus erythematosus (LE) remains less clear and appears to depend on disease subtype. Genetic studies suggest that polymorphisms in MMP and TIMP genes influence both susceptibility to systemic lupus erythematosus (SLE) and its clinical manifestations.

Variants in *MMP-9* and *TIMP-1* have been linked to an increased risk of SLE and to altered *MMP-9* expression. In advanced SLE, lower serum MMP-9 and higher TIMP-1 levels indicate a shift toward proteolytic inhibition, which may promote antigen accumulation when non-selective MMP inhibitors are used [45]. Similarly, *MMP-2* and *TIMP-2* polymorphisms (1575A, 418C) have been associated with elevated transcript and protein levels, reflecting their involvement in inflammatory regulation [46].

MMP-2 and MMP-9 variants correlate with organ-specific complications, including lupus nephritis, hypertension, and cardiovascular disease, while increased serum MMP-3 and urinary MMP-7 levels characterize more severe disease phenotypes [47,48,49,50,51]. However, discrepancies exist: Faber-Elmann et al. (2002) [52] observed elevated MMP-9, but not MMP-2, in SLE serum—mainly in male patients with cutaneous symptoms—whereas Mao et al. (2018) found no such differences [52,53].

In cutaneous lupus erythematosus (CLE), lesional skin shows increased MMP-2, MMP-9, and proMMP-9 expression, with MMP-9 levels correlating with CLASI scores and improving under chloroquine therapy [54,55].

From an immunological perspective, *MMP/TIMP* polymorphisms may affect ECM remodeling, antigen presentation, and chronic inflammation, thereby influencing autoimmune reactivity. Genetic variants in *MMP-2*, *MMP-9*, *TIMP-1*, and *TIMP-2* are potential biomarkers of susceptibility, disease activity, and organ involvement, particularly renal and pulmonary. Monitoring MMP levels—especially alongside inflammatory markers—may assist in stratifying patients and guiding personalized therapeutic approaches in lupus erythematosus.

d. Blistering diseases (pemphigoid and pemphigus)

The initial studies on blistering diseases related to MMPs were conducted by Olkarinen et al. (1983) and showed the presence of MMP-2 and MMP-9 in the skin blisters of patients with bullous pemphigoid (BP) [56]. MMP-9 was first found in the epidermis and endothelial cells [57]. Later research revealed that MMP-9 is located in inflammatory cells such as macrophages, lymphocytes, neutrophils, and eosinophils [58,59,60,61,62], which are abundant in the inflammatory infiltrate of BP blisters. Liu et al. used MMP-9 knockout mice to identify neutrophils as the primary source of active MMP-9 [57], and also showed that MMP-9 interacts with neutrophil elastase (NE), causing epidermal–dermal separation in vivo [63]. Additional experiments by the same team indicated that the plasminogen (Plg) cascade is involved in this process, acting upstream of MMP-9 and leading to the cleavage of the dermo-epidermal junction [64].

Conflicting results were observed when assessing MMP-9’s ability to cleave the extracellular domain of the BP180 autoantigen [63,65]. However, it is well known that MMP-9 can cleave BP180 into tripeptides that markedly enhance neutrophil chemotaxis and NE release in vivo [66]. Therefore, MMP-9 in BP seems to boost the inflammatory response and activate proteolytic enzymes, ultimately resulting in BP180 degradation by NE [67]. Additionally, in vitro, MMP-9 has also been shown to break down type VII collagen and integrin β4 [68].

Inconsistent results were also observed regarding MMP-9 levels in human skin lesions and blister fluid. On one hand, Verraes et al. (2001) reported only the presence of MMP-2/9 proenzymes [59], while Jordan et al. (2023) showed significantly increased levels of the active enzyme [69]. His team further studied IgE antibodies targeting the non-collagenous domain 16A (NC16A) of BP180, which raised MMP-9 levels in mouse skin lesions by triggering its release from eosinophils [69]. Eosinophil extracellular traps (EETosis) are a major source of galectin-10, which boosts MMP production in keratinocytes and fibroblasts, thereby promoting blister formation in BP [70].

Animal models have also been used to study the role of MMP-9 in pemphigus vulgaris (PV), where administration of PV serum to mice resulted in increased MMP-9 expression [71]. Immunolocalization studies of MMP-12 showed its presence in the upper epidermal layers and superficial dermis in PV lesions [72]. Research indicates that inactivating MMP-9 prevents blister formation, but so far, this has only been confirmed in experimental models. The limited number of studies involving BP and PV patients directly prevents a comparison of in vitro findings with human data [73].

e. Alopecia

Alopecia is a skin disorder characterized by disruptions in androgen, cytokine, and growth factor signaling, leading to inhibition of hair follicle growth and differentiation. In vitro studies have demonstrated hair follicle growth arrest and morphological damage driven primarily by epidermal growth factor (EGF) and proinflammatory cytokines such as tumor necrosis factor-α (TNF-α) and interleukin-1α (IL-1α), which partly exert their effects through the stimulation MMPs. Conversely, treatment with TIMPs or their biological analogs promotes hair growth and prevents follicular regression [74].

Jarrousse et al. (2001) [75] demonstrated that human hair follicles primarily produce MMP-2 and MMP-9, localized in the lower inner root sheath (Henle’s layer) of anagen folliclesStimulation with EGF, TNF-α, or IL-1α markedly increased MMP-9 production [75]. In a murine model, Blossom et al. (2007) observed elevated MMP-7 levels following exposure to trichloroacetylaldehyde (TCAH), a metabolite of trichloroethylene (TCE), suggesting that early MMP-7 induction contributes to TCAH-related skin toxicity [76]. Similarly, Heffler et al. (2002) reported a significant rise in MMP-9^+^ CD1a^+^ cells in the dermis during contact sensitization with diphenylcyclopropenone (DPC), a compound with potential therapeutic use in alopecia [77].

The relationship between MMPs and the hair cycle was comprehensively examined by Hou et al. (2016) [78], who found that MMP-2 and MMP-9 mRNA and protein levels increased during anagen and declined during catagen and telogen. In contrast, TIMP-1 and TIMP-2 levels were inversely correlated with MMP-9 and MMP-2, respectively. MMP-2 and TIMP-2 were expressed throughout all follicular structures, while MMP-9 and TIMP-1 were mainly detected in sebaceous glands during all phases and in the inner root sheath during catagen [78].

Collectively, these findings suggest that MMP expression in alopecia is phase-dependent and influenced by external factors, including cytokine milieu and environmental exposures. Although research is limited, current evidence highlights MMP inhibition—particularly targeting MMP-9—as a promising therapeutic avenue for promoting hair regeneration and preventing hair loss.

f. Atopic dermatitis and prurigo nodularis

Atopic dermatitis (AD) is a chronic, recurrent, and heterogeneous inflammatory disorder in which eosinophilia plays a significant role in disease development. Eosinophilia promotes extracellular trap formation and modulates the release of MMPs, suggesting that these proteases may critically influence disease progression. Although eosinophilia is a hallmark of AD, many functional aspects of eosinophils remain unclear. Current data suggest that MMP-8 and MMP-9 may serve as promising diagnostic and predictive biomarkers for AD [79]. This notion is supported by studies measuring various proteases—including ADAM8, ADAM9, MMP-8, Neprilysin/CD10, Cathepsin E, proprotein convertase 9, and urokinase (uPA)—in gingival fluid. The levels of these proteases were lower in patients with moderate to severe AD compared to controls, with differences between groups primarily attributed to MMP-8, Cathepsin E, and ADAM9 [80].

Interestingly, MMP-8 may also influence AD indirectly by modulating cutaneous nerve density, thereby contributing to abnormal itch perception. Studies using cultured neurons from rat dorsal root ganglia demonstrated increased MMP-8 mRNA and protein expression, suggesting a role for MMP-8 in promoting sensory nerve growth within the interstitial collagen matrix through modulation of axonal guidance molecules and/or extracellular matrix components [81]. Moreover, saline skin washings and functional substrate digestion assays revealed significantly higher concentrations of MMP-8 and MMP-9 in AD lesions compared to unaffected skin from AD patients and healthy controls. This proteolytic activity was effectively inhibited by the MMP inhibitor Ro 31-9790. In contrast, AD skin showed lower levels of MMP-10, TIMP-1, and TIMP-2, along with only trace amounts of MMP-1, MMP-3, and TIMP-4 [82]. Elevated MMP-9 levels were also demonstrated by Devillers et al. (2007) [83] and Wang et al. (2024), who further identified the TLR2/TLR1 signaling pathway as the main mechanism by which MMP-9 contributes to inflammation and pruritus in allergic contact dermatitis [84].

Basałygo et al. (2021) [85] reported higher serum concentrations of MMP-1 and MMP-2 in AD patients compared with healthy controls, with no differences in TIMP-1 levels. A positive correlation with EASI was found only for MMP-2 in moderate and severe AD. Interestingly, a higher TIMP-1/MMP-1 ratio was associated with lower transepidermal water loss (TEWL) and improved skin hydration 87. In contrast, De Oliveira Titz et al. (2016) observed no significant differences in serum MMP-2 or TIMP levels, although baseline concentrations of TIMP-1 and TIMP-2 were reduced in cultured eosinophils from AD patients [86]. More recently, Pereira da Fonseca et al. (2024) demonstrated increased MMP-12 activity, predominantly synthesized by M2 macrophages that accumulate within AD lesions. Th2 cytokines and histamine upregulated MMP-12 expression during monocyte differentiation into M2 macrophages, whereas dupilumab treatment effectively blocked this effect [87].

Studies combining genetic and microbiological analyses provided further insight into AD pathogenesis. Wang et al. (2024) [88] evaluated the relationship between microRNA-939 (miR-939) and *Staphylococcus aureus* (*S. aureus*) colonization. They found significantly increased miR-939 expression in keratinocytes after stimulation with heat-killed *S. aureus* (HKSA), as well as in the lesional skin of AD patients. In vitro, miR-939 upregulated *MMP-1*, *MMP-3*, and *MMP-9* expression, while in vivo overexpression of this miRNA enhanced MMP production, facilitating *S. aureus* colonization and aggravating inflammation resembling that observed in AD [88].

Comparative studies of AD and prurigo nodularis (PN) demonstrated markedly higher expression of MMP-1, MMP-3, and MMP-10 in PN lesions compared to AD, along with overall greater MMP activity in PN [88,89]. Moreover, MMP expression was strongly upregulated by oncostatin M (OSM), which is overexpressed in PN lesions, thereby amplifying inflammation through extracellular matrix degradation, immune cell infiltration, cytokine and chemokine activation, and tissue remodeling [82,90,91]. Overall, MMPs contribute significantly to extracellular matrix degradation in PN, promoting nodule formation and engaging neuro-immunological pathways that intensify the itch–scratch cycle—an essential driver of PN pathogenesis [92].

## 3. Inhibition of Metalloproteinases—Therapeutic Perspectives and Research Gaps

- Characteristics of Natural MMP Inhibitors

The balance of proteolytic activity, which allows MMPs to perform their physiological functions, is controlled by TIMPs. TIMPs consist of a family of four members (TIMP-1 to TIMP-4; 22–28 kDa). Any imbalance in the MMP/TIMP ratio causes pathological changes related to ECMbreakdown, leading, for example, to autoimmune disorders [19,93]. Unlike MMPs, which do not show strong selectivity for TIMPs, ADAMs are much more resistant to inhibition [94]. TIMPs inhibit active MMPs by forming tight, non-covalent 1:1 complexes. Furthermore, TIMP-1 and TIMP-2 form specific complexes with latent progelatinases, playing a crucial role in gelatinase activation [93,94]. Transcriptionally, TIMP levels can be regulated, similar to MMPs, by growth factors, cytokines, and chemokines [95]. MMPs also have other endogenous inhibitors, including α_2_-macroglobulin, a cysteine-rich, reversion-inducing protein, and Kazal-motif-containing proteins (RECK), a tissue factor pathway inhibitor, as well as MMP prodomains [96,97]. α_2_-Macroglobulin is a 720 kDa glycoprotein produced in the liver and classified as an acute-phase protein; it acts as an inhibitor of multiple plasma proteases [98,99]. Serifova et al. (2020) [100] demonstrated that α_2_-macroglobulin traps MMP-9 monomers, blocking their proteolytic activity, and upon activation, binds to receptors (e.g., LRP1), enabling internalization and removal of α_2_-macroglobulin/protease complexes from circulation [100]. RECK, a 110 kDa glycoprotein, is involved in tissue remodeling and also limits angiogenesis and metastasis [101]. It has been shown to inhibit MMP-2, MMP-9, MT1-MMP, and ADAM10 [102]. Its functions extend beyond MMP regulation, being vital for the development of blood vessels, collagen fibers, and the basement membrane [99,100,101,102]. Key points regarding MMP inhibitors are summarized in Table 1.

b. Therapies Using Natural MMP Inhibitors and Monoclonal Antibodies

Significant effort has been devoted to developing MMP inhibitors for therapeutic use, including in arthritis (GI168 and tanomastat [115]), cardiovascular diseases (RXP470.1 and batimastat [115,116]), and experimental autoimmune encephalomyelitis (EAE) (ilomastat and D-penicillamine [117,118,119]). However, results from animal models have not consistently translated to human studies. GI168 and tanomastat markedly reduced joint swelling and cartilage as well as bone destruction in preclinical arthritis models [115]. RXP470.1 decreased atherosclerotic plaque areas [116], whereas batimastat, tested in human stents, failed to show clinical benefit [118]. In the EAE model, both ilomastat and D-penicillamine slowed disease progression [117,118], but subsequent clinical trials reported numerous adverse effects [119]. Minocycline, an MMP-9 inhibitor, also limited EAE progression [120,121], while doxycycline, which reduces collagenase activity, has been approved for the treatment of chronic periodontitis [122].

Overall, MMP inhibitors demonstrate promising efficacy in preclinical autoimmune disease models, but their clinical utility remains constrained by a lack of selectivity and the occurrence of adverse reactions. Sub-antibiotic doses of tetracyclines remain among the few approved MMP-targeting options, though they are not typically used as first-line therapy.

A second therapeutic strategy involves monoclonal antibodies (mAbs) that target non-catalytic regions of MMPs. Key mechanisms identified in animal cancer models include induction of apoptosis and inhibition of pancreatic cancer cell migration (GSM-192), suppression of tumor growth and metastasis (DX-2400), reduction in tumor volume (BT1718), mitigation of weight loss and intestinal injury in mouse colitis models (SDS3 and SDS4), and protection against influenza (GM-192, LEM-2/15) [123].

Monoclonal antibodies can selectively inhibit specific MMPs, thereby reducing cell migration, invasion, and metastasis. Similar to natural inhibitors, preclinical results have been encouraging, but clinical success remains limited. Although monoclonal antibody therapies are well established, designing MMP-targeted antibodies is particularly challenging because many fail to inhibit enzymatic activity effectively or cannot distinguish between active and inactive MMP forms [124].

c. Research Gaps in MMP Inhibition Strategies

Effective and safe inhibition of MMPs requires highly selective inhibitors, precise therapeutic targeting, appropriate timing of administration, and consideration of the protective functions of specific MMPs. The inhibitors developed to date have largely been broad-spectrum, blocking multiple MMPs (as well as ADAM/ADAMTS), which has led to adverse effects such as musculoskeletal pain. Therefore, greater specificity toward individual MMPs is necessary, especially given their opposing roles. Importantly, some MMPs perform protective functions in certain diseases. It remains unclear which MMPs are “harmful” and which are “beneficial” in a particular pathological context.

Additionally, MMPs may have opposing roles depending on the stage of disease or infection (e.g., MMP12 is protective early in viral infection but detrimental later). There is a lack of studies that define when and under what conditions MMP inhibition is beneficial versus harmful. Since MMPs are widely expressed across many cell types (epithelial, tumor, immune, fibroblasts), local or targeted drug delivery is likely more effective than systemic blockade. However, optimal methods for controlled delivery and timing are still lacking. Inhibitors targeting non-catalytic domains (e.g., hemopexin domain) often show lower affinity and stability, emphasizing the need for new strategies to enhance their efficacy.

Despite promising results in preclinical models, clinical effectiveness in humans has been limited. Progress will depend on better translational models and predictive biomarkers to identify patients most likely to benefit. Alternative approaches—such as immunotherapy, gene expression inhibition (e.g., RNAi), non-TIMP natural inhibitors (α2-macroglobulin, RECK), and regulatory networks involving TIMPs—remain underexplored and require evaluation for durability and safety compared to traditional inhibitors.

Finally, most studies examine MMPs and TIMPs in isolation, but in vivo, they function within complex regulatory networks. Combining proteomics, transcriptomics, and systems biology approaches is necessary to predict therapeutic outcomes more accurately. The key research gaps include limited selectivity, poor translation from animal models to humans, inadequate understanding of context-dependent MMP functions, and unresolved issues in drug delivery and timing of therapy. Improved biomarkers and innovative strategies (e.g., antibody-based therapies, RNAi, TIMP modulation, protein engineering) are crucial to achieve selective inhibition of harmful MMP activity while maintaining their normal physiological roles.

## 4. Conclusions

MMPs are essential for maintaining tissue homeostasis, and their dysregulation contributes to various diseases, including skin conditions. In inflammatory skin disorders, the relationship between local and systemic MMP activity is complex and varies with each disease. Higher MMP levels are often observed in both affected skin and serum, suggesting a link between ECM remodeling and systemic inflammation. However, these connections are inconsistent—MMP levels in serum do not always correspond to those in the skin. Such differences likely stem from variations in cytokine environments, cell types, and systemic factors like oxidative stress or microbial colonization. Nevertheless, treatments such as anti-TNF-α, chloroquine, or dupilumab frequently restore MMP levels both locally and systemically, indicating a functional relationship. In summary, while MMP activation displays typical inflammatory features across diseases, their local and systemic interactions are influenced by the underlying pathology. Simultaneous assessment of tissue and circulating MMPs in the same patients could help clarify disease mechanisms and support biomarker development. Despite extensive research on MMP inhibitors, findings from animal studies have yet to be applied to humans and cannot currently be used therapeutically. Researchers still encounter conflicting results, likely due to the complex nature of MMPs and their interactions with inhibitors or other regulatory factors. Promising approaches for studying MMP/TIMP include combining proteomics, transcriptomics, and systems modeling to predict therapeutic outcomes better.

## Figures and Tables

**Table 1:** Key findings of studies on endogenous inhibitors in inflammatory diseases.

DISEASE/MODEL	INHIBITOR	STUDY	KEY FINDINGS
Psoriasis	α_2_M	Chodorowska et al., 2004 [103]	elevated α_2_M levels during active disease; returned to normal after successful treatment
Psoriasis	α_2_M	Sikder et al., 2017 [104]	serum α_2_M levels did not differ significantly between controls and patients, either before or after methotrexate treatment
Psoriasis	α_2_M	Schön et al., 2019 [105]	severe psoriasis associated with increased α_2_M
Sclerosis	α_2_M	Freire M et al., 2025 [106]	significant decrease in serum α_2_M in patients exposed to silica
Sclerosis/mouse fibroblasts	RECK	Gutiérrez J et al., 2015 [107]	TGF-β1 reduces RECK expression, promoting β1-integrin activation, fibroblast adhesion, and skin remodeling
Psoriasis	TIMP-1	Michalak-Stoma A et al., 2021 [37]	no differences in TIMP-1 mRNA levels between lesional and non-lesional skin
Psoriasis	TIMP-1	Flisiak et al., 2006 [40]	elevated plasma TIMP-1 levels, no changes in psoriatic scales
SLE	TIMP-2	Brew et al., 2010 [13]	serum and mRNA levels of TIMP-2 significantly increased
SLE	TIMP-1	Vira et al., 2019 [45]	concomitant presence of TIMP-1 372C alleles increased SLE risk; TIMP-1 correlated with SLEDAI score
SLE	TIMP-2	Vira et al., 2020 [46]	active cases: TIMP-1, TIMP-2 significantly elevated (serum and mRNA)
SLE	TIMP-1	Matache et al., 2003 [108]	TIMP-1 secretion is similar to controls
SLE	TIMP-1	Robak et al., 2006 [109]	TIMP-1 levels are lower in SLE; a positive correlation of TIMP-1 correlated with VEGF
CLE	TIMP-1	Ertugrul G et al., 2018 [54]	TIMP-1 increased in lesional skin
Neuropsychiatric SLE	α_2_M	Asano et al., 2017 [110]	increased CSF α_2_M/serum ratio; impaired blood–brain barrier integrity
Lupus nephritis	RECK	Tomita et al., 2025 [111]	miR-6516-3p decreases RECK, increasing MMP-9 expression and renal inflammation
AD exacerbation	TIMP-1	Katoh et al., 2002 [112]	serum TIMP-1 higher than in non-atopic controls; elevated TIMP-1/MMP-3 ratios; correlated with eosinophils, IgE, LDH, eruption score, area, lichenification, and prurigo
AD	TIMP-1	Basałygo et al., 2021 [85]	TIMP-1 > MMP-1 associated with lower TEWL and higher epidermal hydration
AD, eosinophils in vitro	TIMP-1	de Oliveira Titz et al., 2016 [86]	no difference in serum; decreased basal TIMP-1 secretion from unstimulated eosinophils from AD patients
AD	TIMP-1	Lesiak et al., 2010 [55]	increased serum TIMP-1 after chloroquine therapy
Chronic dermatitis model (NC/Kuj mice, Df extract-induced eczema)	TIMP-2	Miyoshi et al., 2005 [113]	TIMP-2 treatment reduced eczema severity, epidermal hyperkeratosis, acanthosis, spongiosis, dermal inflammation; decreased TEWL and epidermal thickness
AD	a_2_-M	Burdina et al., 2014 [114]	elevated levels in AD patients vs. controls; correlated with disease severity (SCORAD)
Pemphigus vulgaris (PV) mouse model	TIMP-3	Cirillo, N et al., 2007 [71]	decreased TIMP-3 in skin of mice injected with PV sera

## Data Availability

No new data were created or analyzed in this study. Data sharing is not applicable to this article.
